# Functional and Evolutionary Characterization of the *CONSTANS* Gene Family in Short-Day Photoperiodic Flowering in Soybean

**DOI:** 10.1371/journal.pone.0085754

**Published:** 2014-01-21

**Authors:** Faqiang Wu, Brian William Price, Waseem Haider, Gabriela Seufferheld, Randall Nelson, Yoshie Hanzawa

**Affiliations:** 1 Department of Crop Sciences, University of Illinois at Urbana-Champaign, Urbana, Illinois, United States of America; 2 USDA-Agricultural Research Service, Soybean/Maize Germplasm, Pathology, and Genetics Research Unit, Department of Crop Sciences, University of Illinois at Urbana-Champaign, Urbana, Illinois, United States of America; Ohio State University, United States of America

## Abstract

*CONSTANS* (*CO*) plays a central role in photoperiodic flowering control of plants. However, much remains unknown about the function of the *CO* gene family in soybean and the molecular mechanisms underlying short-day photoperiodic flowering of soybean. We identified 26 CO homologs (GmCOLs) in the soybean genome, many of them previously unreported. Phylogenic analysis classified GmCOLs into three clades conserved among flowering plants. Two homeologous pairs in Clade I, GmCOL1a/GmCOL1b and GmCOL2a/GmCOL2b, showed the highest sequence similarity to Arabidopsis CO. The mRNA abundance of *GmCOL1a* and *GmCOL1b* exhibited a strong diurnal rhythm under flowering-inductive short days and peaked at dawn, which coincided with the rise of *GmFT5a* expression. In contrast, the mRNA abundance of *GmCOL2a* and *GmCOL2b* was extremely low. Our transgenic study demonstrated that *GmCOL1a*, *GmCOL1b*, *GmCOL2a* and *GmCOL2b* fully complemented the late flowering effect of the *co-1* mutant in Arabidopsis. Together, these results indicate that GmCOL1a and GmCOL1b are potential inducers of flowering in soybean. Our data also indicate rapid regulatory divergence between *GmCOL1a/GmCOL1b* and *GmCOL2a/GmCOL2b* but conservation of their protein function. Dynamic evolution of *GmCOL* regulatory mechanisms may underlie the evolution of photoperiodic signaling in soybean.

## Introduction

Soybean, one of the most important sources of protein and oil in the world, flowers in response to a photoperiod change from long day to short day at the summer solstice [1]. Despite this crop’s agronomic importance, the molecular basis of the photoperiodic flowering response in soybean remains only partially characterized. Several studies have identified eight Maturity Loci, E1 to E8, that affect flowering and seed maturity time [2]–[6]. The causal genes for four of these maturity loci, E1– E4, were recently identified. The photoreceptor gene *PHYTOCHROME A* (*PHYA*) was isolated as the causal gene of E3 and E4 [7], [8], and a homolog of the key flowering gene *GIGANTEA* (*GI*) was isolated as the causal gene of E2 [9]. A transcription factor containing a plant-specific B3 domain was also recently cloned as the causal gene of E1 [10].

In the long-day flowering model plant *Arabidopsis thaliana* (Arabidopsis), the nuclear protein CONSTANS (CO) plays a central role in photoperiodic flowering control [11], [12]. Expression of *CO* mRNA is partly regulated by GIGANTEA (GI), a component of the circadian clock and a regulator of photoperiodic flowering. Under blue light, GI forms a complex with FLAVIN-BINDING, KELCH REPEAT, F-BOX 1 (FKF1) in the late afternoon under long day (LD). On the *CO* promoter, the GI-FKF1 complex degrades CYCLING DOF FACTOR 1 (CDF1), a repressor of *CO* mRNA expression, resulting in activation of *CO* transcription at the end of the day under LD. An example of the “external coincidental mechanism”, CO acts as a point of integration of the internal circadian clock and the external day-night cycles. This integration occurs when *CO* mRNA oscillation coincides with specific light quality at a specific time of the day. In the early morning under LD, the red-light receptor PHYB promotes degradation of CO protein, but the far-red receptor PHYA and the blue-light receptors CRYPTOCHROME 1 (CRY1) and CRY2 stabilize CO protein toward the end of the day. FKF1 is also known to stabilize CO protein in the afternoon by interacting with CO [13]. Other factors, including SUPRESSOR OF PHYA 1 (SPA1), SPA3, SPA4 and CONSTITUTIVE MORPHOGENIC 1 (COP1), regulate CO protein stability during the night. Through the concerted action of light controlling *CO* mRNA oscillation and protein stability, CO protein becomes highly accumulated only at the end of the day under LD. This high CO accumulation activates the floral inducer FT, a mobile florigen signal synthesized in the leaves and transmitted to the shoot apical meristem to cause flowering transition.

Multiple factors regulate *FT* in a CO-independent manner in photoperiodic flowering. For example, GI regulates the amount of the small non-coding RNA *microRNA172* (*miR172*), which induces *FT* expression through repression of APETALA2-like (AP2-like) transcription factors independently of CO [11], [12]. GI also binds directly to the *FT* promoter and enhances *FT* expression. In addition, an Arabidopsis histone deacetylate (HDAC) complex accumulated at dusk under LD is known to modulate histone deacetylation of *FT* chromatin, leading to *FT* expression [14]. Moreover, recent studies suggest that various chromatin modifiers are involved in the control of *FT* expression [15].

The function of CO in flowering induction is conserved among distantly related flowering plants [16]–[18]. In the short-day plant rice, the *HEADING DATE 1* (*Hd1*) gene encoding a CO ortholog induces expression of *FT* homologs *Hd3a* and *RFT1* and promotes flowering under short day (SD), analogous to *FT* induction by CO in Arabidopsis [16]. Similarly, in *Pharbitis nil*, a Japanese morning glory whose short-day flowering habit exemplifies a classical model to study photoperiodism [19], possible involvement of the *Pharbitis* CO homolog (PnCO) and FT homologs (PnFT) in short-day flowering induction has been suggested [20], [21], although unknown factors other than PnCO may induce *PnFT* expression [21]. CO homologs in other species, including potato [22], wheat [23], ryegrass [24], grape [25], alfalfa [26] and barley [27], [28], are also thought to be involved in photoperiodic flowering induction.

Despite the conserved role of CO in flowering, considerable variation in the CO function in flowering has also been reported. Known as a “dual-functional” flowering regulator, rice Hd1 induces flowering under SD but represses flowering under LD. In poplar, ectopically expressed poplar CO1 and CO2, which show highest sequence similarity to Arabidopsis CO among poplar CO homologs, cause changes in metabolic gene expression but no changes in flowering time [29]. Whether poplar recruits other CO homologs or currently unknown genes in flowering remains unclear.

Because of limited information about CO function in soybean, expression analysis has been performed to aid functional characterization of soybean CO homologs (GmCOLs) and FT homologs (GmFTs) [30]–[37]. The function of two of these homologs, GmFT2a and GmFT5a, in flowering control was demonstrated by transgenic approaches in Arabidopsis and soybean [35], [36]. These results suggest a conserved role of GmFTs in flowering in soybean; however, further information is required to clarify the function of GmCOLs and the regulatory interaction between GmCOLs and GmFTs.

Here we report comprehensive characterization of the soybean *CO* gene family and its divergence in gene and protein structure, mRNA expression, regulatory sequence and protein function. Expression of *GmCOL1a* and *GmCOL1b*, which clustered together with Arabidopsis CO in our phylogenetic analysis, exhibited a strong diurnal rhythm under SD and showed a peak at dawn that overlapped with expression of *GmFT5a*. In addition, *GmCOL1a* and *GmCOL1b*, as well as *GmCOL2a* and *GmCOL2b,* complemented the late flowering effect of the *co* mutant in Arabidopsis. Our results support a hypothesis that GmCOL1a and GmCOL1b are key inducers of flowering, likely through the induction of *GmFT5a* in the morning under SD. Our data also indicate dynamic evolution of regulatory sequences of the *GmCOL* family.

## Materials and Methods

### Phylogenetic Analysis

A BLAST (Basic Local Alignment Search Tool) search was carried out using the full-length amino acid sequences of CO and COL homologs from *Arabidopsis*, rice and *Chlamidomonas reinhardtii* against the soybean genome from the Phytozome database (www.phytozome.net). Amino acid sequences similar in length to Arabidopsis CO and COLs were chosen and used for BLAST search for additional CO and COL homologs in the soybean genome. BLAST search continued until no more new homologs appeared. In total, 26 soybean CO homologs (GmCOLs) were obtained. Full-length protein sequences of CO homologs from soybean, Arabidopsis, rice and *Chlamidomonas reinhardtii* were aligned using ClustalW in MEGA5.1 (http://www.megasoftware.net/) with default parameters (pairwise alignment gap opening = 10.0, pairwise alignment gap extension = 0.1, multiple alignment gap opening = 10.0, multiple alignment gap extension = 0.2, and minimum gap separation distance = 4). The phylogenic tree was generated using MEGA5.1 by the Neighbor-Joining method. The bootstrap analysis employed 2,000 replicates.

### Plant Growth Condition and Sampling

Seeds of the seven soybean genotypes were provided by the USDA Soybean Germplasm Collection. Two common North American cultivars, Clark (PI 548533) and Williams 82 (PI 518671); the four near isogenic lines (NILs) of E loci carrying contrasting alleles in E1, E2, E3 and E5 (PI 547431; PI 547432; PI 547610; PI 591490); and *Glycine soja* (PI 549046) were used (Table 1). Plants were grown in the greenhouse under SD (10 hours of light, 6∶45–16∶45) and LD (16 hours of light, 6∶45–22∶45) conditions at 25°C and were sampled every four hours at six time points, T1– T6 (6∶30, 10∶30, 14∶30, 18∶30, 22∶30 and 2∶30), over a 24-hour time period three weeks after germination. For a shift experiment, plants were first grown under LD for three weeks and then transferred to SD for 5 days. A whole shoot above the cotyledon, including 3–4 trifoliates, stem and shoot meristems, was harvested from each plant. Three to four biological replications were sampled for each time point and photoperiod condition.

**Table 1:** Seven soybean inbred lines, their allele types for Maturity Loci (E loci) and the days to flowering.

Variety	Maturity Loci(E loci)	Days to flowering		
		Field	Greenhouse	
			SD	LD
Clark	e1E2E3E4e5E7	33	27±1	63±2
Williams 82	E2	33	27±1	61±3
L65-3366	E1E2E3E4e5E7	61	29±2	97±10
L66-432	e1e2E3E4e5E7	50	29±1	74±4
L74-441	e1E2e3E4e5E7	56	30±1	52±3
L92-1195	e1E2e3E4E5E7	36	28±0	70±9
*Glycine soja*	N/A	N/A	27±1	107±8

The E loci genotype information is based on the record in the USDA soybean germplasm collection. The flowering time in the field is the average of flowering time data from two consecutive years based on the record in the USDA soybean germplasm collection.

### RNA Preparation

Samples stored in −80°C were first disrupted in a mortar for homogenization and then ground by the TissueLyser II (Qiagen®) while frozen. The fine powder was then used for RNA preparation following the protocol of the RNeasy® Plant Mini Kit (Qiagen®) with a few minor modifications. The RNA samples were measured for quality and quantity using the NanoDrop™ 1000 Spectrophotometer (Thermo Scientific®), and cDNA libraries were synthesized using SuperScript™ III First-Strand Synthesis System for RT-PCR kit (Invitrogen™). The quality of the cDNA was tested by RT-PCR using a pair of primers specific for the housekeeping gene *GmPBB2* [30] (Table S1).

### Quantitative RT-PCR

The quantitative RT-PCR (qRT-PCR) was performed using a 7900 HT Fast Real-Time PCR System (Applied Biosystems®), following the manufacturer’s manual of FastStart Universal SYBR Green Master (Rox) (Roche Applied Science®). Gene-specific primers used for qRT-PCR are listed in Table S1. All reactions were carried out in a 384-well Clear Optical Reaction Plate (Applied Biosystems®), with a volume of 15 µl per well that, consisted of 7.5 µl 2×FastStart Master Mix, 6.4 µl sterilized distilled water, 0.3 µl of each primer (10 M), and 0.5 µl template. The resulting data were recorded and analyzed by the 7900 HT RT-PCR System software. Transcript levels were calculated relative to those of the reference gene *GmPBB2* (Table S1). Each qRT-PCR reaction was performed in triplicate and all data were presented as means ± SEM.

### Transcriptome Sequencing Data Analysis

189 RNA samples, 3 photoperiod treatments (SD, LD, and a shift from LD to SD), 3 time points (6∶30, 14∶30, and 22∶30), 7 genotypes (Table 1) and 3 biological replications were used for RNA sequencing. Quality of RNA was tested using electrophoresis on a 1% agarose gel, and 5 ug total RNA for each sample was submitted to the W. M. Keck Center for Comparative and Functional Genomics in the Roy J. Carver Biotechnology Center at the University of Illinois in Urbana-Champaign. The cDNA libraries were prepared using Illumina's TruSeq RNAseq Sample Prep kit, and RNA sequencing was performed using Illumina sequencing technology with 12 lanes. Samples were assigned randomly to each lane in order to reduce the effect of lanes. To increase the number of reads per sample, we allocated fewer samples to each lane (15 or 16 samples per lane) than the maximum possible number of samples. Obtained reads were 100 nt in length. Number of reads ranged from 150 million to 185 million per lane and 9.5 million to 12.3 million per sample. This will result in approximately 175–228 reads per gene if all genes are equally expressed. Data quality was ensured by FastX (http://hannonlab.cshl.edu/fastx_toolkit/index.html) and fastQC (http://www.bioinformatics.bbsrc.ac.uk/projects/download.html#fastqc).

The Bowtie package [38] was used to map and align the reads to the soybean transcriptome (Williams 82) obtained from the Phytozome database [39]. The latest 54,175 predicted protein coding loci and their 73,320 transcripts were used. Counts of aligned read were obtained by using an in-house python script. Normalization of mapped reads was performed by RPKM (Reads Per Kilobase per Million mapped reads) [40]. The RNA sequencing data (the accession numbers GSM1234545 – GSM1234733) were deposited in the Gene Expression Omnibus (GEO) site at the National Canter for Biotechnology Information (NCBI) website (http://www.ncbi.nlm.nih.gov/geo/).

### Motif Logo Analysis, Dot Plot Analysis and Hierarchical Clustering Analysis

For the motif logo diagrams, sequences corresponding to representative motifs were aligned and then submitted to Weblogo [41] for visualization. The dot plot was created using Nucleic Acid Dot Plots program (http://www.vivo.colostate.edu/molkit/dnadot/index.html) with the following parameters: window size: 11; mismatch limit: 0. Coding sequence and 2.5 kb upstream non-coding region (introns were excluded) were used for generating the diagram. Hierarchical clustering analysis was performed using MeV software [42]. The hierarchical clustering method [43] was used to construct a binary tree by grouping expression of *GmCOL*s in RPKM values obtained by RNA sequencing.

### Gene Cloning, Vector Construction and Arabidopsis Transformation

DNA fragments containing the coding sequence of soybean *COL1a*, *COL1b*, *COL2a* and *COL2b* were amplified from cDNA of the soybean Williams 82 using gene-specific primers (Table S1) and then cloned into the pCR2.1 vector (Invitrogen) following manufacturer’s instructions. Several independent clones were sequenced for validation purposes. The coding sequence of the four genes was subcloned into the entry vector pCR8 (Invitrogen) and finally transferred to the overexpression binary vector pEarley100 [44] via the LR Gateway recombination reaction following manufacturer’s instructions (Invitrogen). Arabidopsis plants (Col-0 wild type and *co-1* mutant introgressed into Col-0 by outcrossing four times) were infected with *Agrobacterium tumefaciens* strain pGV3101 transformed with the obtained pEarley100 clones using the floral dipping method [45]. For screening T1 tranformants, T1 seeds obtained from the dipped plants were grown on soil and sprayed with Basta (glufosinate ammonium, Sigma, 100 mg/L) at least three times.

### Arabidopsis Growth Conditions and Measurement of Flowering Time

Arabidopsis plants were grown in growth chamber under LD conditions (16 h light/8 h dark) with white fluorescence light at 22°C. For flowering time determination, the number of rosette leaves was counted at the time the first flowers bloomed [46].

## Results

### Identification of CO Homologs in Soybean

**Figure 1:**
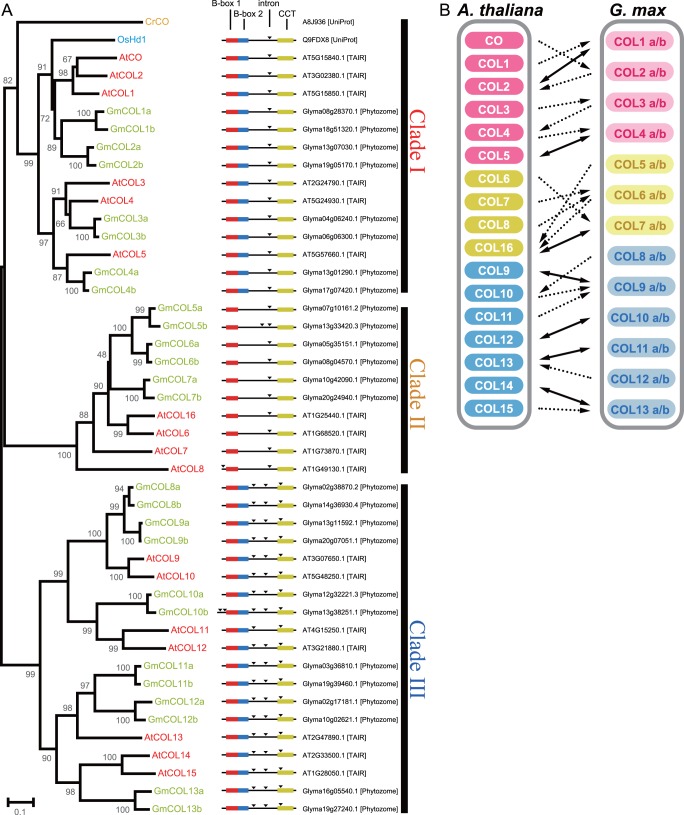
CO homologs in soybean. (A) Phylogenetic analysis of full-length amino acid sequences of CO homologs of soybean (Gm), Arabidopsis (At), rice (Os) and *Chlamydomonas reinhardtii* (Cr). Numbers at nodes indicate the value of 1,000 bootstrap analyses. The domain structure of B-box 1 (red rectangles), B-box 2 (blue rectangles), CCT (yellow rectangles) and the introns (black triangle) of the genes are shown. Followed are the accession numbers of the proteins and their corresponding databases (in square brackets). (B) BLAST best hits of CO homologs in Arabidopsis (*A. thaliana*) and soybean (*G. max*). The BLAST best hits are shown by dotted lines with an arrow head, and the protein pairs of the reciprocal best hits are shown by lines with double-head arrows.

A BLAST (Basic Local Alignment Search Tool) search using the full-length amino acid sequences of CO and COL homologs from Arabidopsis, rice and *Chlamydomonas reinhardtii* identified 26 soybean CO homologs (GmCOLs). Phylogenetic analysis clarified the evolutionary relationships of GmCOLs (Figure 1A). Each GmCOL was then assigned a name based on its homology level to Arabidopsis CO and COLs, with a designation of a or b for homeologous pairs originating from the most recent duplication event (e.g. GmCOL1a and GmCOL1b). The 26 GmCOLs comprised 13 such homeologous pairs that were classified into three clades: 4 pairs in Clade I, three pairs in Clade II, and six pairs in Clade III. Clade I contained two sub-clades that nested two such homeologous pairs, and Clade III contained two sub-clades that nested three such homeologous pairs. This observation corresponds well with the multiple genome duplication events that have occurred in soybean [39]. Among the eight GmCOLs in Clade I, GmCOL1a, GmCOL1b, GmCOL2a and GmCOL2b clustered together with Arabidopsis CO and rice CO (Hd1), well-characterized flowering inducers.

CO homologs, which constitute a subgroup of zinc finger proteins, contain one or more zinc-binding B-box domains at the N terminus and a CCT domain at the C terminus [47], [48]. CO homologs in each clade possess specific gene structure and protein domains [48], [49]. GmCOL homologs in each clade showed structural characteristics similar to each other (Figure 1A). GmCOLs in Clades I and II contained one intron, with the exception of GmCOL5b, which contained three introns. GmCOLs in Clade III contained three introns. GmCOLs in Clades I and III possessed two B-boxes and a CCT domain in the protein structure, whereas GmCOLs in Clade II possessed only one B-box domain and a CCT domain. Comparison of the amino acid sequences of the domains indicated that B-box 2 domain was less conserved compared to B-box 1 and CCT domains between Arabidopsis and soybean (Figure S1).

A reciprocal BLAST search then identified potential orthologous relationships in CO homologs between Arabidopsis and soybean (Figure 1B). Supporting the observed clades being conserved among species in the phylogenetic analysis, the BLAST best hits of all Arabidopsis CO homologs were identified from soybean homologs within the same clade and vice versa. The analysis revealed seven reciprocal BLAST best hit pairs: 2 in Clade I, 1 in Clade II and 4 in Clade III. GmCOL2a and GmCOL2b were the BLAST best hits of Arabidopsis CO; however, no reciprocal BLAST best hit pairs existed between Arabidopsis CO and GmCOLs.

### Photoperiods Affect Flowering Time Variation

All seven genotypes used in this study flowered earlier under SD than under LD. *G. soja* flowered extremely late under LD (106.7 days after germination) but as early as other genotypes under SD (27.2 days), exhibiting the strongest response to photoperiods among the genotypes (Table 1). Flowering time among the genotypes showed large variation under LD (51.5 days to 106.7 days), whereas flowering time under SD showed more subtle variation (27.2 days to 30.2 days).

### Photoperiodic Regulation of *GmCOL*s

**Figure 2:**
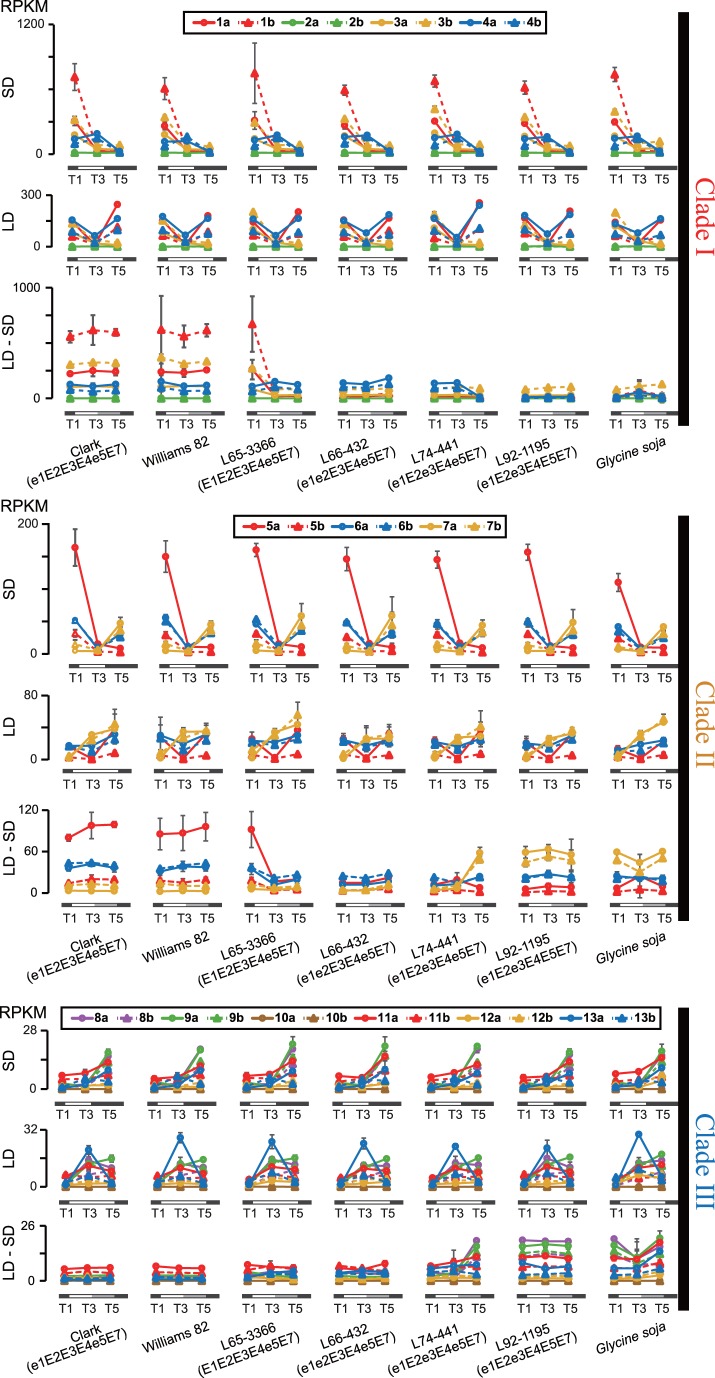
mRNA abundance of *GmCOL*s measured by RNA sequencing. RPKM values are displayed on the left. The top panel shows *GmCOL*s in Clade I, the middle panel shows *GmCOL*s in Clade II, and the bottom panel shows *GmCOL*s in Clade III. SD is 10 hours light (6∶45–16∶45), LD is 16 hours light (6∶45–22∶45), and LD-SD is a shift from three weeks LD to 5 days SD. Samples are three representative time points: T1 (6∶30), T3 (14∶30) and T5 (22∶30).

To survey the mRNA abundance of *GmCOLs* among different genotypes and photoperiods, an RNA sequencing approach was employed with three representative time points: T1 (6∶30), T3 (2∶30) and T5 (22∶30) (Figure 2). A wide range of mRNA abundance was observed among *GmCOL*s. *GmCOL1a, GmCOL1b, GmCOL3b* and *GmCOL5a* showed notably high expression with strong rhythmic patterns particularly under SD with a peak at T1 (6∶30), while *GmCOL2a, GmCOL2b, GmCOL10a* and *GmCOL10b* displayed faint expression under both SD and LD. Expression patterns of homeologous genes were generally well conserved. All expressed *GmCOL*s showed different expression patterns between SD and LD, suggesting photoperiodic regulation of their mRNA abundance.

Under SD, the peak expression levels of *GmCOL1a, GmCOL1b, GmCOL3b* and *GmCOL5a* at T1 (6∶30) exceeded 250 RPKM (Reads Per Kilobase of transcript per Million mapped reads), with *GmCOL1b* showing the highest level of approximately 700 RPKM. Under LD, the expression levels of these genes at T1 (6∶30) were more than 2-fold lower than under SD, with *GmCOL1b* showing the strongest reduction of more than 7-fold. *GmCOL2a* and *GmCOL2b* demonstrated much lower expression (below 2.0 RPKM) than *GmCOL1a* and *GmCOL1b* under both SD and LD.

### Variation in Expression of *GmCOL*s among Genotypes

No significant variation in the mRNA abundance of *GmCOL*s appeared among the seven genotypes at the representative time points based on the RNA sequencing data under SD and LD (Figure 2). In contrast, striking variation appeared among the genotypes in the shift experiment. *GmCOL1a, GmCOL1b* and *GmCOL3b* in Clade I showed elevated mRNA abundance at all three time points in Clark and Williams 82 and at T1 (6∶30) in a NIL, L65-3366 (E1E2E3E4e5E7), in response to the photoperiod shift. A very similar response in mRNA abundance to the photoperiod shift was observed in *GmCOL5a* in Clade II. Another notable variation among genotypes was the elevated mRNA abundance of *GmCOL7a, GmCOL7b* and many *GmCOL*s in Clade III at T3 (22∶30) in L74-441 (e1E2e3E4e5E7) and at all three time points in L92-1195 (e1E2e3E4E5E7) and *G. soja*.

### Clustering of *GmCOL* Expression Patterns

**Figure 3:**
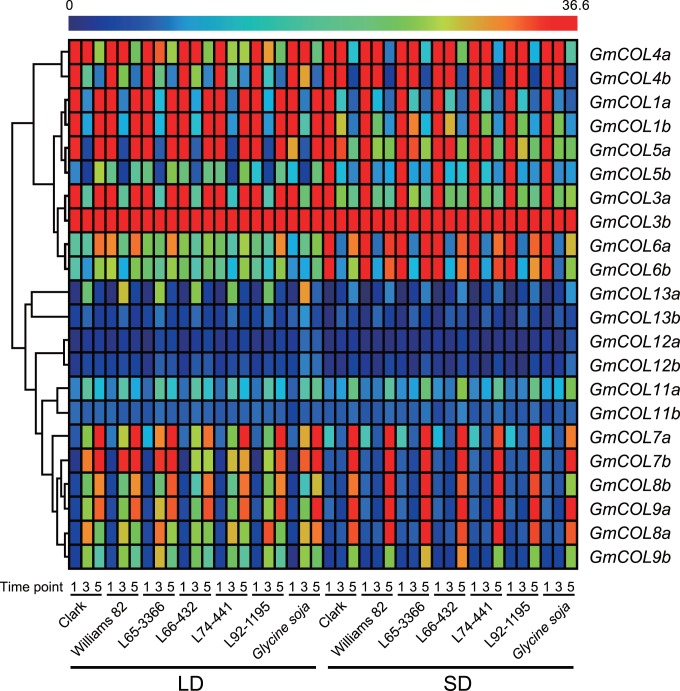
Hierarchical clustering of *GmCOL*s based on their mRNA abundance under LD and SD. *GmCOL2a*, *GmCOL2b*, *GmCOL10a* and *GmCOL10b* are excluded due to their extreme low abundance. To demonstrate the variation in the mRNA abundance, a rainbow color scheme was used in which red indicates high expression and blue indicates low expression.

A hierarchical clustering analysis of the mRNA expression patterns of *GmCOL*s under LD and SD revealed rapid divergence of regulatory mechanisms among *GmCOL*s as well as high conservation among recently derived homeologs. Two main branches appeared: a cluster of *GmCOL*s from Clades I and II, and a cluster of *GmCOL*s from Clade III (Figure 3), in agreement with the coding sequence similarity noted in the phylogenetic analysis (Figure 1A). An exception was *GmCOL7a* and *GmCOL7b* from Clade II clustering together with *GmCOL8a*/*GmCOL8b* and *GmCOL9a*/*GmCOL9b* from Clade III, creating a sub-clade nested under the cluster of Clade III genes (Figure 3). This observation is consistent with the similarity in their mRNA abundance in response to the photoperiod shift (Figure 2). All pairs of homeologous genes derived from the last genome duplication clustered together, except the *GmCOL7a/mCOL7b, GmCOL8a/GmCOL8b* and *GmCOL9a/GmCOL9b* pairs (Figure 3).

### *GmCOL1a* and *GmCOL1b* Show a Diurnal Rhythm with a Peak before Dawn under SD

**Figure 4:**
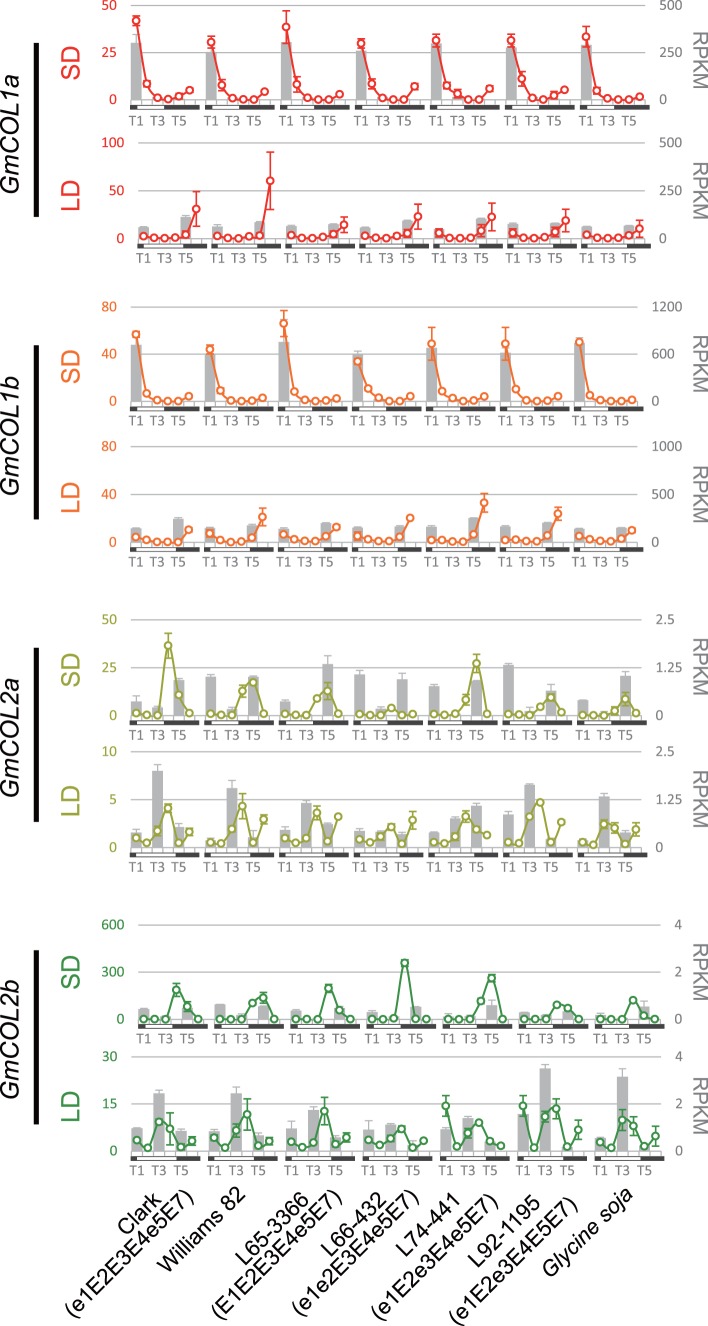
mRNA abundance of *GmCOL1a, GmCOL1b, GmCOL2a* and *GmCOL2b*. Circles and solid lines represent the data obtained by qRT-PCR, and gray bars represent the data obtained by RNA sequencing. Relative expression levels of qRT-PCR among the samples in each panel are shown on the left, and RPKM values of RNA sequencing are shown on the right. SD is 10 hours light (6∶45–16∶45) and LD is 16 hours light (6∶45–22∶45). Time points T1–T6 represent 6∶30, 10∶30, 14∶30, 18∶30, 22∶30 and 2∶30. Note that RNA sequencing samples contain three representative time points: T1, T3 and T5.

*GmCOL1a, GmCOL1b, GmCOL2a* and *GmCOL2b* mRNA accumulation was further characterized by qRT-PCR at all six time points. The diurnal expression patterns of *GmCOL1a* and *GmCOL1b* observed by qRT-PCR corresponded very well with the RNA sequencing results (Figure 4). However, *GmCOL2a* and *GmCOL2b* showed less conserved patterns between RNA sequencing and qRT-PCR, likely due to their extreme low abundance.

*GmCOL1a* and *GmCOL1b* demonstrated similar expression patterns to each other, with a diurnal rhythm in both SD and LD (Figure 4). Under SD, *GmCOL1a* and *GmCOL1b* expression rose in late night (T6: 2:30), peaked at dawn (T1: 6:30) and declined sharply over the next four hours (T2: 10:30). The abundance remained low during the day and after dusk until late night. Under LD, *GmCOL1a* and *GmCOL1b* expression remained low during the day and early evening, peaked in the middle of the night (T6: 2:30) and declined before dawn. In general, expression patterns of *GmCOL1a* and *GmCOL1b* demonstrated similarity among the seven genotypes under SD in general. Notable variations among the genotypes, however, appeared in LD: higher peak expression of *GmCOL1a* at T6 (2∶30) in Clark and Williams 82 than in other genotypes, and higher expression of *GmCOL1b* at T6 (2∶30) in a near isogenic line (NIL), L74-441 (e1E2e3E4e5E7).

*GmCOL2a* and *GmCOL2b* exhibited a similar diurnal expression rhythm under SD, but their rhythmic expression patterns were less clear under LD (Figure 4). In clear contrast to *GmCOL1a* and *GmCOL1b*, expression of *GmCOL2a* and *GmCOL2b* in SD peaked after dusk (T4: 18:30) and declined during the night. Under LD, their expression appeared to peak at two time points: T4 (18∶30) and T6 (2∶30).

### *GmCOL1a* and *GmCOL1b* Coincide with *GmFT5a* under SD

**Figure 5:**
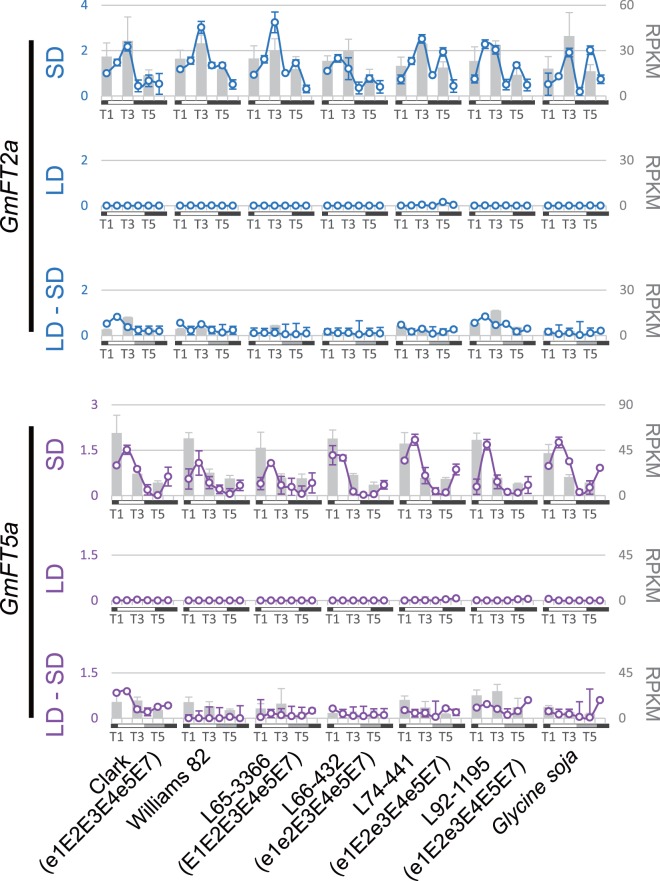
mRNA abundance of *GmFT2a* and *GmFT5a*. Circles and solid lines represent the data obtained by qRT-PCR, and gray bars represent the data obtained by RNA sequencing. Relative expression levels of qRT-PCR among the samples in each panel are shown on the left, and RPKM values of RNA sequencing are shown on the right. SD is 10 hours light (6∶45–16∶45), LD is 16 hours light (6∶45–22∶45) and LD-SD is a shift from three weeks LD to 5 days SD. Time points T1–T6 represent 6∶30, 10∶30, 14∶30, 18∶30, 22∶30 and 2∶30. Note that RNA sequencing samples contain three representative time points: T1, T3 and T5.

Abundance of *GmFT2a* and *GmFT5a* mRNA accumulation peaks at 3–4 hours after dawn under SD [33]. Similarly, our RNA sequencing and qRT-PCR results indicated that *GmFT5a* expression rose in late night (T6: 2:30), peaked at dawn (T1: 6:30) and declined sharply over the next four hours (T2: 10:30) under SD, whereas no expression was detected under LD (Figure 5). Peak expression of *GmFT5a* occurred at T2 (10∶30) in all genotypes except L66-432 (e1e2E3E4e5E7), in which *GmFT5a* peaked at T1 (6∶30). The mRNA abundance of *GmFT5a* corresponds very well with that of *GmCOL1a* and *GmCOL1b,* which peaked at dawn under SD preceding the *GmFT5a* peak (Figures 4 and 5).

Like *GmFT5a, GmFT2a* showed expression under SD but not under LD. However, the expression pattern of *GmFT2a* under SD appeared different from that of *GmFT5a*: it peaked at T3 (14∶30), declined at T4 (18∶30) and increased again at T5 (22∶30). The second peak was more apparent in L65-3366 (E1E2E3E4e5E7), L74-441 (e1E2e3E4e5E7), L92-1195 (e1E2e3E4E5E7) and *G. soja* than in other genotypes. This second peak of *GmFT2a* seems to overlap with the peak expression of *GmCOL2a* and *GmCOL2b* (Figures 4 and 5).

### Rapid Divergence of *GmCOL* Regulatory Mechanisms

**Figure 6:**
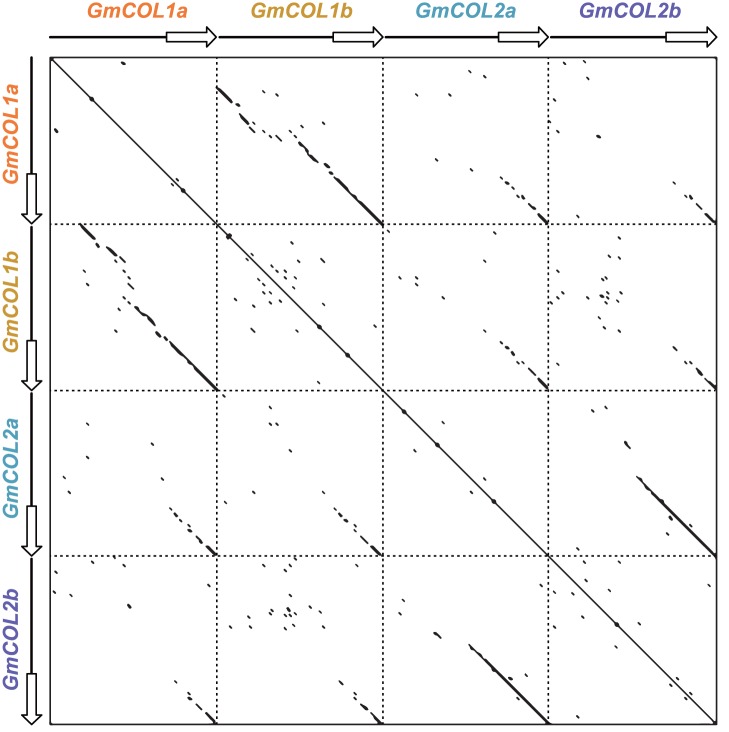
Comparison of the 2.5*GmCOL1a*, *GmCOL1b*, *GmCOL2a* and *GmCOL2b*. Empty arrows indicate the coding sequence (introns were excluded); lines indicate the upstream intergenic region. Conserved sequences between two sequences are evident from the detected diagonal dotted lines. The dot plot was created using the Nucleic Acid Dot Plots program, with the following parameters: window size: 11; mismatch limit: 0.

Comparison of the 2.5 kb upstream intergenic region of *GmCOL1a*, *GmCOL1b*, *GmCOL2a* and *GmCOL2b* showed highly conserved promoter sequences of homeologous genes derived from the last genome duplication (Figure 6). *GmCOL1a* and *GmCOL1b* shared high sequence similarity throughout the coding region and the 2.5 kb upstream intergenic region. Similarly, *GmCOL2a* and *GmCOL2b* displayed high similarity in the coding region and a restricted upstream intergenic region (0.5 kb). In contrast, comparison between homeologous genes derived from the earlier genome duplication, *GmCOL1a/GmCOL1b* and *GmCOL2a/GmCOL2b*, showed less or no obvious similarity in the coding region and the upstream intergenic region, which is consistent with their diverse mRNA expression patterns. Other *GmCOL*s demonstrated similar trends in sequence divergence of the upstream intergenic region: high similarity between homeologous gene pairs derived from the last genome duplication and no similarity among homeologous genes derived from the earlier genome duplication (data not shown), due to the longer evolutionary time.

Despite the striking similarity observed in the mRNA abundance of *GmCOL5a* and *GmCOL1a/GmCOL1b* in response to photoperiods (Figure 3), no sequence similarity was observed in the upstream intergenic region (data not shown). Similarly, no sequence similarity was observed in the upstream intergenic region among *GmCOL7a*/*GmCOL7b*, *GmCOL8a*/*GmCOL8b* and *GmCOL9a*/*GmCOL9b*.

### GmCOL1a, GmCOL1b, GmCOL2a and GmCOL2b Share Conserved Protein Function

**Figure 7:**
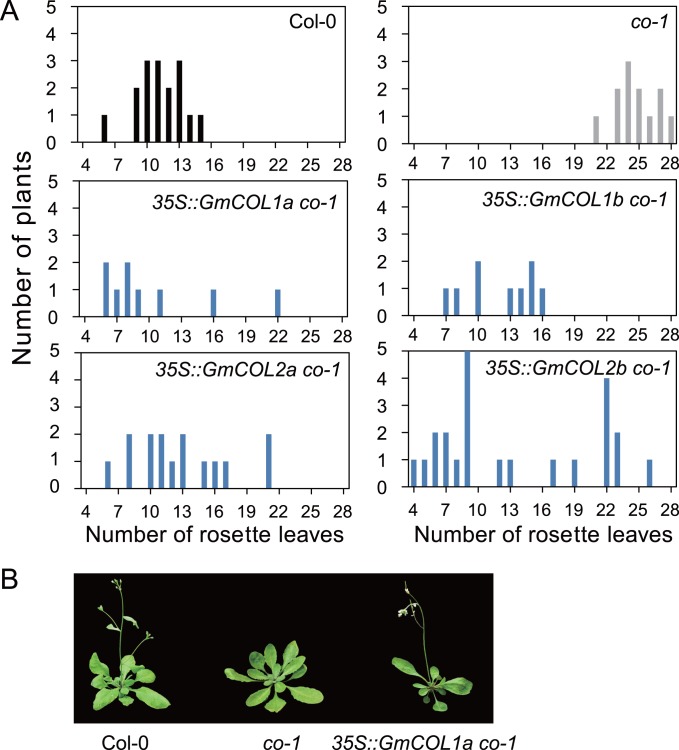
Overexpression of GmCOL1a, GmCOL1b, GmCOL2a and GmCOL2b rescued the late flowering phenotype of Arabidopsis *co-1* mutant. (A) The vertical axis indicates the number of T1 transgenic plants, and the horizontal axis indicates the number of rosette leaves. (B) Col-0 (left), *co-1* mutant (middle) and *co-1* mutant overexpressing GmCOL1a (right).

In marked contrast to *GmCOL*’s rapid evolution of regulatory sequences, their functional evolution is slower. To examine the protein function of GmCOL1a, GmCOL1b, GmCOL2a and GmCOL2b derived from the past genomic duplication events, we expressed the coding sequences of *GmCOL1a*, *GmCOL1b*, *GmCOL2a* and *GmCOL2b* under the control of the CaMV *35S* promoter in Arabidopsis plants carrying a mutation in the *CO* gene. While the *co-1* mutant showed significant late flowering compared to wild-type, the transgenic plants expressing *GmCOL1a*, *GmCOL1b*, *GmCOL2a* and *GmCOL2b* fully complemented the late flowering effect of *co-1* (Figure 7). This observation suggests that the protein function of GmCOL1a, GmCOL1b, GmCOL2a and GmCOL2b was highly conserved, despite the significant regulatory divergence between *GmCOL1a*/*GmCOL1b* and *GmCOL2a*/*GmCOL2b.*

## Discussion

### GmCOL1a and GmCOL1b are Potential Flowering Inducers

We identified 26 COL homologs from the soybean genome and characterized their photoperiod response in mRNA accumulation, sequence divergence and protein function. The *CO* gene family participates in a wide range of events in plant development in response to photoperiodic signaling, including flowering [50], seedling growth [51], [52], dormancy [53] and tuberization [54] in vascular plants, as well as cell growth in the green algae *Chlamidomonas* [18]. Functional divergence of the *CO* gene family in Arabidopsis has been reported. For instance, despite their high sequence similarity with CO, COL1 and COL2 cause no changes in flowering time when ectopically expressed [55]. COL3 influences root growth and lateral root formation [51] and acts also as a repressor of flowering, unlike CO. Similar to COL3, COL9 represses flowering [56]. We observed that the soybean *CO* gene family, like its counterpart in Arabidopsis, showed highly diverse expression patterns while responding to photoperiods, strongly suggesting GmCOLs’ functional divergence in multiple aspects of soybean photoperiodic response, including flowering.

We gathered evidence indicating that homeologous GmCOL1a and GmCOL1b are inducers of flowering in soybean. A key piece of evidence supporting the role of GmCOL1a and GmCOL1b in flowering is that they complemented the function of Arabidopsis CO in our transgenic study (Figure 7). Further evidence for the role of GmCOL1a and GmCOL1b in flowering stems from their high sequence similarity to Arabidopsis CO (Figure 1A). Our phylogenetic analysis showed that two homeologous pairs of GmCOLs, GmCOL1a/GmCOL1b and GmCOL2a/GmCOL2b, clustered together with Arabidopsis CO and rice Hd1. Among *GmCOL*s, *GmCOL1a* and *GmCOL1b* showed the highest expression, while *GmCOL2a* and *GmCOL2b* showed extremely low expression and a weak peak after dusk (Figure 4), indicating the possibility of *GmCOL2a* and *GmCOL2b* becoming pseudogenes or evolving novel function.

Offering more support for their role in flowering, *GmCOL1a* and *GmCOL1b* display strong photoperiod responsiveness in two ways. First, the mRNA abundance level and patterns of *GmCOL1a* and *GmCOL1b* showed striking discrepancies between SD and LD, with a high peak under flowering-inductive SD (Figure 4). Second, *GmCOL1a* and *GmCOL1b,* as well as *GmCOL3a* and *GmCOL5a,* exhibited a strong response to the photoperiod shift (Figure 2). Taken together, our results suggest that *GmCOL1a* and *GmCOL1b* are inducers of photoperiodic flowering. Our work may also indicate that photoperiodic flowering regulation by the *CO* gene family in flowering plants stems from an ancestral gene of Clade I that harbors Arabidopsis CO and rice Hd1 as well as GmCOL1a and GmCOL1b.

### GmCOL1a and GmCOL1b are Potential Activators of *GmFT*s

Our expression study showed that the peak expression of *GmCOL1a* and *GmCOL1b* at dawn overlapped with the peak expression of *GmFT5a* in the morning under flowering inductive SD (Figures 4 and 5). This result may indicate that the regulatory interaction between CO and FT reported in Arabidopsis, rice and other flowering plants is conserved in soybean. However, further clarification is required to confirm this hypothesis.

In the SD flowering plants rice and Pharbitis, expression of *FT* homologs peaks at the end of the night under SD, consistent with SD flowering plants’ requirement of exposure to long nights. Our results and published findings show that *GmCOL1a* and *GmCOL1b* expression peaks at the end of the night when *GmFT5a* expression rises (Figures 4 and 5), indicating that a mechanism similar to the one in rice and Pharbitis measures photoperiod to regulate flowering in soybean.

In contrast, *GmFT2a* expression peaks several hours after dawn in the afternoon, unlike the peak expression of *GmFT5a* in the morning. The time lag between the peak expression of *GmCOL*s and *GmFT2a* may suggest that unidentified mechanisms are necessary for *GmFT2a* induction. Such mechanisms may include soybean GI homologs. In Arabidopsis, GI is known to induce *FT* expression independently of CO [11], [12]. Similarly, the *GI* homolog *LATE1* in pea regulates the pea *FT* homolog *FTL* but has only minor effect on the expression of a pea *CO* homolog [57]. Supporting this hypothesis, we observed that *GmGIa*, the causal gene of the maturity locus E2, peaked at T3 (14∶30), corresponding well with the peak expression of *GmFT2a* (Figure S2). In addition, *GmFT2a* expression was considerably low in the NIL L66-432 (e1e2E3E4e5E7) that carried the recessive e2 allele and showed lower accumulation of *GmGIa* at T3 (14∶30) (Figures 5 and S2).

Another clear difference between *GmFT2a* and *GmFT5a* is that *GmFT2a* exhibits a second expression peak during the night under SD (Figure 5), which coincides with the peak expression of *GmCOL2a* and *GmCOL2b* (Figures 4 and 5). The role of the *GmFT2a* second peak in flowering regulation and the possible involvement of *GmCOL2a* and *GmCOL2b* in *GmFT2a* induction are currently unclear.

### Accelerated Regulatory Evolution of *GmCOL*s

How gene function diversifies following gene duplication is a major question in evolutionary biology. It has been suggested that regulatory divergence, as well as protein functional divergence, is the primary evolutionary source of novel gene function [58]–[60]. Our results show that regulatory sequence divergence among *GmCOL1a/GmCOL1b* and *GmCOL2a/GmCOL2b* preceded their protein functional divergence (Figures 4, 6 and 7), supporting the importance of regulatory divergence that may lead to functional evolution of duplicated genes. Providing further insight into regulatory evolution of the *GmCOL* gene family, the mRNA accumulation of *GmCOL5a*, which belongs to Clade II, showed a photoperiodic response resembling that of *GmCOL1a* and *GmCOL1b* in Clade I (Figure 2). The striking similarity in the photoperiodic regulation of mRNA accumulation among distantly related *GmCOL1a*/*GmCOL1b* and *GmCOL5a* may indicate rewiring of genetic networks through evolution of co-regulation mechanisms among them. Similarly, remarkable resemblance observed in the mRNA abundance of *GmCOL7a*/*GmCOL7b* in Clade II and *GmCOL*s in Clade III may also imply that regulatory evolution leads to re-arrangement of genetic interactions and, hence, evolution of novel function. Although further study is required to clarify their regulatory interaction and function, our work demonstrates dynamic evolution of the soybean *COL* genes and their regulatory mechanisms that may underlie the evolution of the photoperiodic signaling in soybean.

## Supporting Information

Figure S1 **Comparison of B-box 1, B-box 2 and CCT domains of COL homologs.** Amino acid sequences of B-box 1, B-box 2 and CCT domains of COL homologs in Clades I, II and III in Arabidopsis (At) and soybean (Gm) are compared. Conserved amino acids are shown in large characters for visualization created by Weblogo (Crooks et al., 2004). CO homologs in Clade II do not contain B-box 2.
(DOCX)

Figure S2 **mRNA abundance of** ***Glyma06g23026/E1, Glyma10g36600/E2/GmGIa, Glyma19g41210/E3/GmPHYA3*** **and** ***Glyma20g22160/E4/GmPHYA2*** **measured by RNA sequencing.** RPKM values are displayed on the left. SD is 10 hours light (6∶45–16∶45), LD is 16 hours light (6∶45–22∶45), and LD-SD is a shift from three weeks LD to 5 days SD. Samples are three representative time points: T1 (6∶30), T3 (14∶30) and T5 (22∶30).
(DOCX)

Table S1 **Primers used for this work.**
(XLSX)
