# Supplementary material for: Functional and Evolutionary Characterization of the CONSTANS Gene Family in Short-Day Photoperiodic Flowering in Soybean
Source: PLoS One. 2014 Jan 21;9(1):e85754. doi: 10.1371/journal.pone.0085754 (PMC3897488; doi:10.1371/journal.pone.0085754)
Supplement: Figure S1 — Comparison of B-box 1, B-box 2 and CCT domains of COL homologs. Amino acid sequences of B-box 1, B-box 2 and CCT domains of COL homologs in Clades I, II and III in Arabidopsis (At) and soybean (Gm) are compared. Conserved amino acids are shown in large characters for visualization created by Weblogo (Crooks et al., 2004). CO homologs in Clade II do not contain B-box 2. (DOCX) [file pone.0085754.s001.docx]

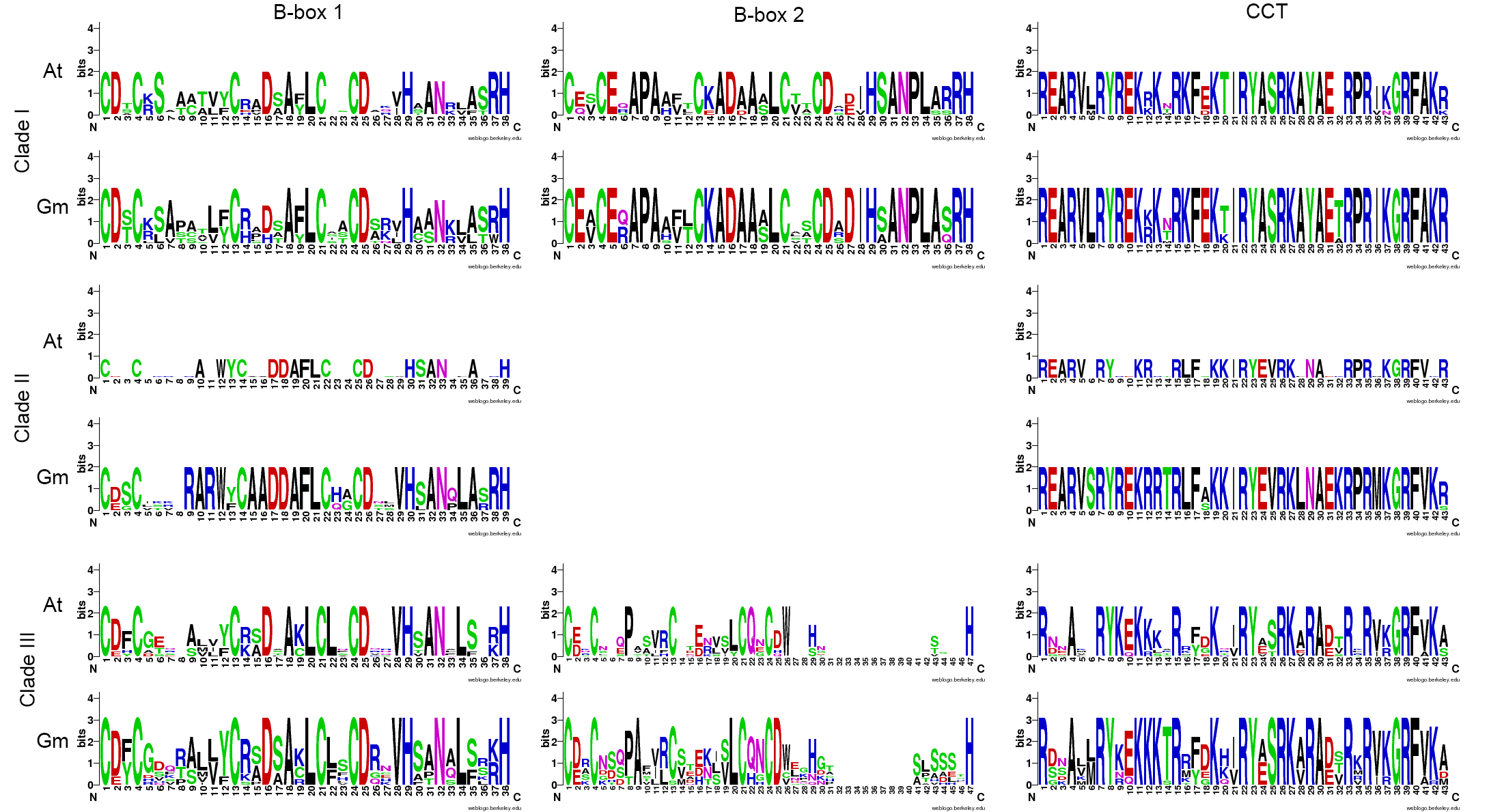


Figure S1. Comparison of amino acid sequences of B-box 1, B-box 2 and CCT domains of COL homologs in Clades I, II and III in Arabidopsis (At) and soybean (Gm). Conserved amino acids are shown in large characters for visualization created by Weblogo (Crooks et al., 2004). CO homologs in Clade II do not contain B-box 2
