# Supplementary material for: Functional and Evolutionary Characterization of the CONSTANS Gene Family in Short-Day Photoperiodic Flowering in Soybean
Source: PLoS One. 2014 Jan 21;9(1):e85754. doi: 10.1371/journal.pone.0085754 (PMC3897488; doi:10.1371/journal.pone.0085754)
Supplement: Figure S2 — mRNA abundance of Glyma06g23026/E1, Glyma10g36600/E2/GmGIa, Glyma19g41210/E3/GmPHYA3 and Glyma20g22160/E4/GmPHYA2 measured by RNA sequencing. RPKM values are displayed on the left. SD is 10 hours light (6∶45–16∶45), LD is 16 hours light (6∶45–22∶45), and LD-SD is a shift from three weeks LD to 5 days SD. Samples are three representative time points: T1 (6∶30), T3 (14∶30) and T5 (22∶30). (DOCX) [file pone.0085754.s002.docx]

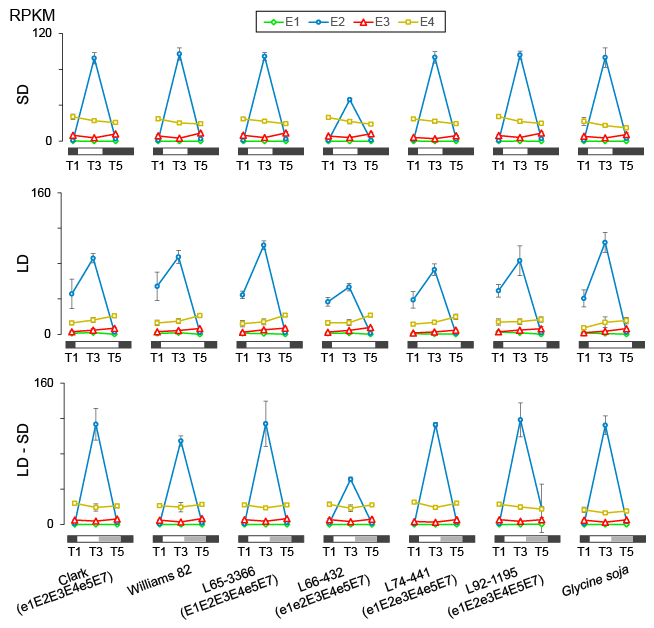


Figure S2. mRNA abundance of *Glyma06g23026/E1, Glyma10g36600/E2/GmGIa, Glyma19g41210/E3/GmPHYA3* and *Glyma20g22160/E4/GmPHYA2* measured by RNA sequencing. RPKM values are displayed on the left. SD is 10 hours light (6:45-16:45), LD is 16 hours light (6:45-22:45), and LD-SD is a shift from three weeks LD to 5 days SD. Samples are three representative time points: T1 (6:30), T3 (14:30) and T5 (22:30).
